# Water Stress Differentially Modulates the Expression of Tomato Cell Wall Metabolism-Related Genes in *Meloidogyne incognita* Feeding Sites

**DOI:** 10.3389/fpls.2022.817185

**Published:** 2022-04-15

**Authors:** Pasqua Veronico, Laura Cristina Rosso, Maria Teresa Melillo, Elena Fanelli, Francesca De Luca, Aurelio Ciancio, Mariantonietta Colagiero, Isabella Pentimone

**Affiliations:** Istituto per la Protezione Sostenibile delle Piante, Consiglio Nazionale delle Ricerche, Bari, Italy

**Keywords:** water stress, *Meloidogyne incognita*, tomato, feeding site, cell wall, transcriptome

## Abstract

Microscopic observations and transcriptomic RNA-Seq analyses were applied to investigate the effect of water stress during the formation of tomato galls formation 1 and 2 weeks after inoculation with the root-knot nematode *Meloidogyne incognita*. Water stress affected root growth and the nematode ability to mount an efficient parasitism. The effects of water stress on the feeding site development were already observed at 1 week after nematode inoculation, with smaller giant cells, delayed development, and thinner cell walls. These features suggested changes in the expression levels of genes involved in the feeding site formation and maintenance. Gene Ontology (GO) enrichment and expression patterns were used to characterize differentially expressed genes. Water stress modified the expression profile of genes involved in the synthesis, degradation, and remodeling of the cell wall during the development of nematode feeding site. A comparison of gene expression with unstressed galls revealed that water stress intensified the up or downregulation of most genes. However, it particularly influenced the expression pattern of *expansin A11* (Solyc04g081870.4.1), *expansin-like B1*(Solyc08g077910.3.1), a *pectin acetylesterase* (Solyc08g005800.4.1), and the *pectin methylesterase pmeu1* (Solyc03g123630.4.1) which were upregulated in unstressed galls and repressed by water stress, at both sampling times. The expression of most members of the genes involved in cell wall metabolism, i.e., those coding for Csl, fasciclin, and COBRA proteins, were negatively influenced. Interestingly, alteration in the expression profiles of most dirigent protein genes (DIRs) and upregulation of five gene coding for Casparian strip domain protein (CASP)-like proteins were found. Gene expression analysis of galls from water stressed plants allowed us to better understand the molecular basis of *M. incognita* parasitism in tomato. Specific genes, including those involved in regulation of cellulose synthesis and lignification process, require further study to develop defense strategies against root-knot nematodes.

## Introduction

Plants are continuously exposed to a broad range of environmental stresses during their entire life cycle. Stresses include both abiotic factors, such as drought, salinity, heat, or cold, and biotic factors, such as bacteria, viruses, fungi, or nematodes. Both types of stress can have a devastating impact on plant growth and yield under field conditions (Suzuki et al., 2014). Plants developed a complex morphological and molecular response system to prevent and/or tolerate stress damages and survive (Lamers et al., 2020). Most efficient mechanisms involve the recognition of stress-related features (either chemical or physical) by dedicated receptors and the transduction and the propagation of the signal by downstream players, resulting in cellular responses characterized by an alteration of the expression of a variety of genes. The response of plants to simultaneous biotic and abiotic stresses is distinct for individual stresses, and not merely additive (Atkinson and Urwin, 2012; Ramegowda and Senthyl-Kumar, 2015). For example, the combination of water deficit and plant-parasitic nematodes is a realistic threat under field conditions and could drastically impact crop productivity. Drought stress itself generates physiological changes in higher plants, including loss of turgor, osmotic adjustment, and reduced leaf water potential (Le Gall et al., 2015). These changes are associated with increases in endogenous abscisic acid (ABA), which plays a role in regulating drought stress responses in plants (Schachtman and Goodger, 2008; Cutler et al., 2010). On the other hand, nematode infection can exacerbate or counteract the effects of water stress on plants, as root parasitism greatly influences the plant-water relations (Atkinson et al., 2013).

Root-knot nematodes *Meloidogyne* spp. have an intimate relationship with their hosts as they derive nutrients directly from living root cells. Therefore, factors that alter plant growth, such as water shortage, may also alter the root-nematode interaction.

Motile second stage juveniles (J2) of root-knot nematodes penetrate roots in a region behind tips, along the elongation zone. By migrating intercellularly, they reach the differentiating vascular cylinder. Here, the invading nematodes select some parenchyma cells where they induce and maintain, with their secretions, a specialized feeding site (FS). Selected root cells then differentiate into hypertrophied, multinucleate, and metabolically active giant cells (GCs) (Gheysen and Mitchum, 2011). GCs serve as an exclusive food source for the nematode to develop into a reproductive female. Symptoms of root infection include proliferation of the cells surrounding GCs, consequent vascular alterations, hypertrophy of endodermis, and cortex that ultimately generate swollen roots tissues known as galls (Gheysen and Mitchum, 2011). At the cellular level, the changes that occur during the formation of the nematode FS are complex and include hormonal metabolism adjustment, transcription factors modulation, cytoskeleton rearrangement, and modifications of the cell wall metabolism (Shukla et al., 2018). Cell wall metabolism includes cell wall biogenesis and cell wall remodeling. In nematode FS, the cell wall undergoes complex modifications involving a reorganization of the wall extension and various synthesis and degradation-related processes (Sobczak et al., 2011).

The cell wall plays a fundamental role in plant defense as it provides structural support during development and represents the first line of defense against pathogens, parasites, and abiotic stressors including drought (Underwood, 2012; Ricardi et al., 2014).

Information provided by different omics data analyses and functional characterization of individual genes in response to drought showed a modulation of several genes involved in cell wall metabolism. Modulation of cellulose synthesis in Arabidopsis (Chen et al., 2005) and the overexpression of expansin genes in several plant species (Guo et al., 2011; Xu et al., 2014; Liu et al., 2019) lead to improved drought tolerance and higher survival rates. Similar findings were found with other genes, i.e., *pectin methylesterase* (Wormit and Usadel, 2018) or *xyloglucan endotransglucosylase/hydrolase* (XTH), in transgenic Arabidopsis and tomato, respectively (Cho et al., 2006; Choi et al., 2011).

Tomato (*Solanum lycopersicon*) represents an excellent model to study the host–nematode interactions due to the availability of routinely well-annotated genome reference. Moreover, despite its large-scale cultivation and industrial value, it is still sensitive to severe stresses of abiotic and biotic nature (Kissoudis et al., 2016). In the present study, we evaluated how water stress impacts *Meloidogyne incognita* parasitism in tomato. This knowledge could be highly informative in plant protection due to the importance of root-knot nematodes as pests and to the effects of climate change that is expected to alter precipitation regimes toward more frequent and severe drought events. To date, few studies have been carried out on transcriptome analyses under combined abiotic and biotic stresses. By using microscopic observation of GCs and in depth transcriptomic (RNA-Seq) analyses, we show here that water stress affects the ability of nematodes to mount an efficient parasitism by limiting the FS development and nematode reproduction. Additionally, we describe differentially expressed genes (DEGs) responsive to combined stresses and genes mainly involved in cell wall modifications during GC development in galls of normally watered and water-stressed plants.

## Materials and Methods

### Plants, Growth Conditions, and Water Stress Treatment

Seeds of tomato (*Solanum lycopersicum* cv. ‘San Marzano nano’) were kept for germination in quartz sand in a growth chamber at 25°C and then transplanted to 10 ml pots containing soil-sand mixture (3:1). The seedlings were grown in a growth chamber under controlled conditions at 25°C, with a light intensity of 150 μmol/m^2^/s and a 16 h:8 h light/dark cycle and watered with Long Ashton solution containing 300 μM phosphate. After 20 days, 40 plants were transferred in plastic pots filled with 800 g of sandy soil and watered with filtered tap water (twice a week) and Long Ashton solution (once a week). After 20 days, the plants were divided into four groups: (i) regularly watered (treatment “C,” control plants), (ii) regularly irrigated and to be inoculated with *M. incognita* J2 (treatment “RKN”), (iii) subjected to a water stress (treatment “WS”), and (iv) subjected to water stress and to be inoculated with *M. incognita* (treatment “RKN_WS”). Ten replicates for each group have been used and arranged in a randomized block design. The experiment was carried out according to Balestrini et al. (2019). Before treatments, the pots were weighed. Plants from the groups C and RKN were regularly watered throughout the entire experimental period (Supplementary Figure 1A), whereas those to be stressed (WS and RKN_WS) were not watered until they showed stress symptoms, i.e., leaves were folded up due to loss of turgidity, if pots weighted about 200 g less than their initial weight, and/or a loss previously described to be needed to reach a moderate water stress condition (Volpe et al., 2018). From this moment, the plants received the amounts of water or nutritive solution needed to keep their last weight in order to maintain a moderate stress level (Supplementary Figure 1B).

### Nematode Infection Assay

A pure population of the root-knot nematode *M. incognita* race 2 was multiplied on tomato (cv. Roma) in a growth chamber. Egg masses were hand-picked from infested roots and kept for hatching in water in a growth chamber at 25°C. Freshly hatched J2 were used for inoculation.

Each plant from the RKN and RKN_WS groups was inoculated with 1,200 freshly hatched J2 according to the scheme reported in Supplementary Figures 1A,B. The plants were maintained in a growth chamber at 25°C. Plants from all treatments were harvested 62 and 69 days after sowing [corresponding to 7- and 14-days post-nematode inoculation (dpi) for RKN and RKN_WS treatments]. Soil was washed from roots, and the height and the fresh root and shoot weights were subsequently measured. Nematode infection was evaluated by counting the number of galls per plant with a stereomicroscope.

### Morphological and Morphometric Analyses

Galls collected at 7 and 14 dpi from normally watered (RKN) and water-stressed (RKN_WS) roots were hand-dissected under a stereomicroscope and fixed in a mixture of 1.5% glutaraldehyde and 3% paraformaldehyde (Sigma-Aldrich), dehydrated in an ethanol series, and embedded in acrylic resin LR White (Sigma, St. Louis, MO, United States) (Melillo et al., 2014). Cell structures, i.e., nuclei and cell walls, were visualized by toluidine blue staining. Embedded galls were cut in serial cross sections (2.5 μm thick) through their length, then stained briefly with 1% toluidine blue in 1% borax solution and mounted in Depex. The sections of 30 galls from three different plants (ten galls per plants), both from normally watered and water-stressed roots at 7 and 14 dpi, were observed using Leica DM 4,500 B light microscope (Leica Microsystems, Milan, Italy). The FS images were recorded by a Leica DFC 450C camera. Gall diameters were measured by using the line tool provided by the ImageJ basic package^1^. The two largest GCs were selected for each FS at 7 and 14 dpi, and their area was measured with ImageJ. The average size area and standard error for a minimum of 50 GCs were also measured from the gall sections at each time point.

Cellulose distribution was visualized by Calcofluor white staining. The dye was prepared by adding 100 μl of stock solution (FLUKA, Sigma-Aldrich; Calcofluor white M2R 1 g L^–1^, Evans blue 0.5 g L^–1^) to 1 ml of water. Evans blue in the solution allows to quench background fluorescence. The sections (2.5 μm thick) were stained in this solution for 5 min at room temperature and protected by light, washed with water, and mounted in glycerol. Samples were observed with a Leica DM 4500 B using an A filter cube (excitation filter: 340–380 nm and detection filter LP 430 nm).

### RNA Extraction, RNA-Seq Library Preparation, and Sequencing

For RNA-Seq experiments, visible galls and corresponding portions of roots from non-inoculated plants were collected. For C, RKN, WS, and RKN_WS samples, each plant was considered as a biological replicate. For each sample and collection time (7 and 14 days/dpi), two independent biological replicates were performed, except for WS and RKN_WS at 7 days/dpi where three replicates were considered due to a lower amount of developed roots. Samples were immediately stored at −80°C until further use. Root tissues (100 mg) were powdered in liquid nitrogen. Total RNA was extracted and purified using the RNeasy Plant Mini Kit (Qiagen, Milan, Italy) following the manufacturer’s protocol. RNA quantity and quality was determined with a Nanodrop 2,000 spectrophotometer (Thermo Fisher Scientific Inc., Wilmington, DE, United States) and a Bioanalyzer 2,100 (Agilent Technologies, Santa Clara, CA, United States). cDNA libraries preparation and sequencing were performed by Identity Governance and Administration (IGA) Technology Services (Udine, Italy). The libraries were sequenced on an Illumina HiScanSQ, generating single reads 75 nt in length.

### RNA-Seq Data Analysis and Differential Gene Expression Quantification

Raw sequences were processed for quality check using the ‘‘RNA-seq analysis’’ functions included in CLC Genomics Workbench software v.10.1 (QIAGEN, Aarhus, Denmark^2^). Adapters, indexes, and genomic sequences added during the sequencing process were removed. Filtered reads from each sample were then aligned to the reference genome of *S. lycopersicum* (Genome version SL4.0 and Annotation ITAG4.0^3^) using CLC (similarity parameter = 0.8; identity parameter = 0.8; mismatch/insertion/deletion penalties = 2/3/3; multi-position matches allowed) and employed to quantify the abundance of all tomato transcripts, measured as Reads Per Kilobase Million (RPKM) (Mortazavi et al., 2008) and Transcripts Per Million (TPM) (Wagner et al., 2012). A multiple correlation test (Pearson’s correlation) on RPKM values for all pairwise combinations was performed for preliminary batch comparisons of replicates and experimental conditions. Principal Component Analysis (PCA) was also performed to analyze the variation sources in the dataset. Differential expression analysis was performed with the CLC statistical tools, applying the Generalized Linear Model (GLM) which corrects differences in library size between the samples, over-dispersion caused by biological variability, and the effects of confounding factors. Differentially expressed genes (DEGs) were evaluated at each time points (7 and 14 dpi) comparing the RPKM expression values of every gene for each sample group against the reference group (normally watered uninoculated roots, treatment C). The gene expression level was considered significant when it displayed at least a two-fold change (Fc), with a *p*-value ≤ 0.05 and TPM value ≥ 5. The terms “upregulation” and “downregulation” were used to indicate transcript expression levels (Fc ≥ 2 or Fc ≤ −2, respectively) higher or lower than those observed in the reference. The DEGs were then submitted to functional analysis. Heatmaps were constructed with TPM mean values using online available tools^4^ (Supplementary Table 1).

### Functional Analysis of Tomato Differentially Expressed Genes

Enrichment analysis was performed using AgriGO ver.2.0^5^ (Tian et al., 2017) in order to identify, in each DEG selected set (RKN_WS vs. C at 7 and 14 dpi), the over-represented GO categories. Through enrichment analysis, molecular functions and biological processes over-represented with a statistical significance (Fisher’s Exact Test: *p*-value ≤ 0.05; Hochberg FDR ≤ 0.05) were considered. The *S. lycopersicum* cDNA libraries ITAG3.2 and ITAG4.0 version^6^ were used as reference. The Venn/Euler diagram online tool^7^ was used to show DEGs that were unique and common for each condition.

Identification of cell wall metabolism-related DEGs was carried out by comparing RKN_WS transcriptome with a local cell wall database created by consulting available *S. lycopersicum* UniProtKB^8^ and SolGenomics^9^ databases. Used terms were as follows: cell wall organization or biogenesis, cell wall biosynthesis, and plant cell structures.

### cDNA Synthesis and Real-Time Quantitative PCR Analyses

The expression of 10 DEGs (Supplementary Table 2) involved in stress response and cell wall metabolism was analyzed using quantitative reverse transcription-PCR (RT-qPCR) to validate the RNA-Seq results. Plant tissue collection and RNA extraction were performed as previously described. cDNA was synthesized from 0.5 mg of DNase-treated RNA using oligo(dT) primers and a reverse transcription kit (Promega Corporation, Madison, WI, United States).

Real-time PCR assays were performed on an Aria device (Agilent Technologies Inc., Santa Clara, CA, United States) in 20-μl reaction mixtures containing 0.4 μM of each gene-specific primer, 10 μl of GoTaq qPCR Master Mix (Promega), and 1 μl (50 ng) of template cDNA. Specific qRT-PCR primers were designed on available sequences of *S. lycopersicum* SL4.0 (see Text Footnote 3) using Primer3 and an online basic local alignment search tool (BLAST)^10^ (Supplementary Table 2). The amplification specificity was confirmed by a single peak in the dissociation curve at the end of the PCR and a single band in agarose gel electrophoresis. Gene expression was calculated using AriaMx version 1.6 software and expressed as a relative quantitation to the normalizer gene actin (BT013524).

### Statistical Analyses

Data were subjected to analysis of variance (ANOVA) and significant differences among treatments were analyzed by applying a least-significant difference test (*p* < 0.05) for morphometric parameters and Turkey’s pairwise test (*p* < 0.05) for RT-qPCR validation data. Values between treatments and untreated and uninfected control were compared at each time point. The number of galls per plant and the GC area measurements at 14 dpi were statistically analyzed by Student’s *t*-test (*p* < 0.01). Statistical analyses were performed using Plot IT software version 3.20i and Past 4.03 (Hammer et al., 2001). Box plot graphics were produced with R using library *ggplot2* ver. 3.3.3 (RStudio, PBC version 4.0.5).

## Results

### Effects of Water Stress on Tomato Growth and Pathogenesis Rating

In this study, tomato plants were firstly subjected to a moderate water stress before root-knot nematode infection. Plants from group RKN_WS (water stressed and infected) were inoculated with *M. incognita* J2, and sampling was performed at 7 and 14 dpi. Plants from group WS (water stressed) were harvested 7 and 14 days after they reached the condition of moderate water stress. Plants from groups C (normally watered and uninfected) and RKN (normally watered and infected) were regularly watered along the experimental period (Supplementary Figures 1A,B). The aim was to understand the water stress-related responses in roots infested by *M. incognita*. Comparisons of WS vs. C plants and RKN_WS vs. RKN plants showed that water stress significantly (*p* < 0.05) decreased tomato plant heights at both times (Table 1). Likewise, shoot fresh weight was significantly reduced by water stress both in WS and RKN_WS plants compared to regular watering (C and RKN) (Table 1). Water stress affected root growth, particularly at 14 days, when a severe weight reduction (by 73%) was observed in WS plants compared to C (Table 1). The effect of nematode parasitism (RKN vs. C) on fresh root weight was clearly appreciable due to the presence of galls on RKN plants (Table 1). Water stress affected nematode infection, causing a severe decrease in the number of galls per plant (RKN_WS vs. RKN; *p* < 0.01) both at 7 and 14 dpi (around −84%) (Table 1). Moreover, a significant reduction in the diameter of galls both at 7 and 14 dpi was observed in RKN_WS as compared with RKN (Table 1).

**TABLE 1:** Effect of water stress on tomato vegetative growth and *M. incognita* infection.

Recording time	Treatments	Height (cm)	Fresh shoot weight (g)	Fresh root weight (g)	Galls/plant	Gall diam. (μ m)	GC area (μ m^2^)
7 days	C	30.83 ± 0.31^b^	20.95 ± 1.48^b^	3.33 ± 0.47^ab^			
	RKN	31.67 ± 0.80^b^	24.49 ± 0.36^c^	5.81 ± 0.27^c^	209 ± 22	245 ± 9	10462 ± 903
	WS	22.67 ± 0.80^a^	10.53 ± 0.57^a^	2.47 ± 0.09^a^			
	RKN_WS	21.25 ± 0.73^a^	10.36 ± 0.35^a^	3.15 ± 0.20^a^	34 ± 3**	176 ± 6**	5247 ± 290**
14 days	C	32.50 ± 1.15^b^	24.74 ± 2.41^b^	6.52 ± 0.52^b^			
	RKN	35.00 ± 2.13^b^	27.21 ± 2.01^b^	8.59 ± 0.63^c^	276 ± 17	253 ± 14	24071 ± 1319
	WS	20.00 ± 0.73^a^	5.63 ± 0.56^a^	1.75 ± 0.05^a^			
	RKN_WS	20.17 ± 1.01^a^	5.96 ± 0.50^a^	2.14 ± 0.12^a^	46 ± 10**	208 ± 11*	10297 ± 658**

*Data recorded 7 and 14 days after nematode infection.*

C, normally watered and uninfected; RKN, unstressed and M. incognita infected; WS, water-stressed; RKN_WS, water-stressed and M. incognita infected plants. Values are means from six replicates ± SD. Same letters represent values that are not significantly different according to Duncan’s Multiple Range Test (p < 0.05). Asterisks *, ** indicate significant differences according to Student’s t-test p < 0.05 or < 0.01 respectively.

### Nematode Feeding Site Development

Morphological changes in GC development were analyzed in RKN_WS plants and compared to those in RKN roots. Detailed observations were carried out on serial cross sections of galls at 7 and 14 dpi. Measurements of the GC area revealed that the cells in RKN_WS were consistently less expanded than in RKN roots both at 7 and 14 dpi (−50 and −57%, respectively; *p* < 0.01) (Table 1).

**FIGURE 1:**
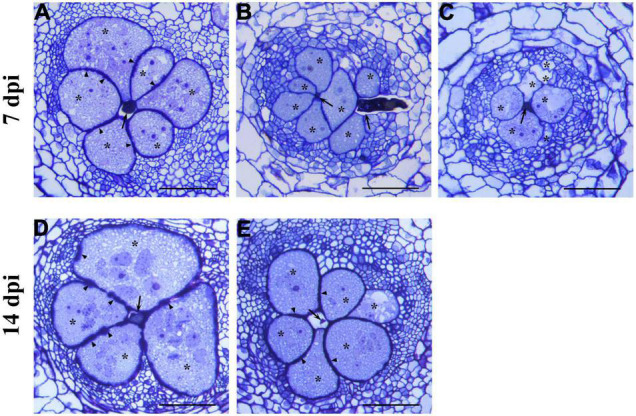
Histological analysis of giant cells (GCs) induced by *Meloidogyne incognita* in normally watered (RKN) and water-stressed (RKN_WS) tomato roots at 7- and 14-day post inoculation (dpi). Cross sections (2.5 μm) were stained with toluidine blue and observed at light microscope. **(A)** Well developed and metabolically active giant cells in RKN roots at 7 dpi. **(B,C)** Feeding sites in RKN_WS galls at 7 dpi. **(B)** GCs look similar to those in unstressed gall but are smaller in size. **(C)** GCs contain large vacuoles and cytoplasm poorly active showing a delay in their development. Cell walls in all RKN_WS feeding sites at 7 dpi are thinner than those in RKN galls. **(D)** GCs in RKN galls at 14 dpi show a large expansion with increase in cell wall thickness and density of cytoplasm. **(E)** GCs in RKN_WS galls at 14 dpi look significantly smaller but similar in appearance to GCs in unstressed galls. *, GCs; arrow, nematode; arrowhead, thickened cell walls; scale bar, 100 μm.

Histological observations were performed to monitor possible alterations in the development of the nematode FS. At 7 dpi, the RKN galls showed large multinucleate GCs mostly occupying the vascular cylinder area (Figure 1A). They showed a conspicuous number of nuclei, several small vacuoles, and a dense, granular, metabolically active cytoplasm. Unevenly thickened cell walls could also be noted (Figure 1A). At 14 dpi (Figure 1D), the vascular cylinder was entirely occupied by GCs with uniformly dense and granular cytoplasm and scarce vacuolation, allowing the provision of nutrients for nematode development. Cell wall thickening was observed in all GCs. Numerous sections of RKN_WS galls at 7 dpi consistently showed that many GCs had an appearance comparable with that of unstressed GCs, although they were significantly smaller (Figure 1B). A number of GCs showed large vacuoles typical of the early infection and poorly active cytoplasm, revealing a significant delay in development (Figure 1C). In all observed GCs, the cell walls were thinner than those in RKN galls. At 14 dpi, the GCs in RKN_WS galls were shown to have dense cytoplasm and thickened walls, a similar feature to those in unstressed galls though their size remained considerably smaller (Figure 1E).

### RNA-Seq and Transcriptomic Profiles of Tomato Galls in Response to Water Stress

Sequencing analyses were performed on 7 and 14 dpi galls from RKN and RKN_WS tomato plants vs. the respective C plants. The aim was to gain insights into the molecular mechanisms that are affected by water stress within the nematode FS.

Over 220 million high quality 75-bp single reads were generated across all sampled roots at 7 dpi and about 174 million at 14 dpi (ranging from 38 up to 65 million per sample) (Supplementary Table 3). The sequences were deposited in FASTQ format in the Short Read Archive (SRA) of National Centre for Biotechnology Information (NCBI) and are available under BioProject accession PRJNA734743.

In total, 70–94% of good-quality reads were mapped onto the *S. lycopersicum* genome (SL4.0 assembly) across all samples (Supplementary Table 3). A high Pearson’s correlation coefficient (r) was observed between RPKM values for each set of sample replicates sequenced (average *r* = 0.94 at 7 dpi and *r* = 0.90 at 14 dpi).

**FIGURE 2:**
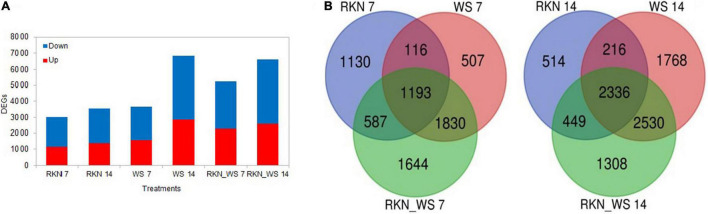
Differentially expressed genes (DEGs) in normally watered and *M. incognita* infected (RKN), water-stressed (WS) and water-stressed and *M. incognita* infected plants (RKN_WS), compared to uninfected and normally watered control (C) (Fold change ≥ 2 or ≤–2, *p*-value ≤ 0.05 and TPM ≥ 5). **(A)** Number of DEGs for each treatment and sampling time. Bars represent total number of up (red) and downregulated (blue) genes compared to the respective C at 7 and 14 dpi. **(B)** Venn diagrams showing exclusive and common DEGs for each experimental condition.

The percent of DEGs relative to the reference tomato transcriptome (34.075 protein coding genes in ITAG4.0) evaluated at each time points (7 and 14 dpi) increased for stressed plants in the sampling time and, when compared to the reference normally watered, uninfected roots (treatment C). This fraction increased after 14 days, raising from 9, 11, and 15% at 7 dpi to 10, 20, and 19% at 14 dpi for RKN, WS, and RKN_WS, respectively. For each treatment, downregulated transcripts always exceeded the upregulated ones (Figure 2A).

Comparative analysis across treatments showed that 37, 14, and 31% of DEGs at 7 dpi and 15, 26, and 20% at 14 dpi were exclusive for treatments RKN, WS, and RKN_WS, respectively. The percentage of DEGs common to all conditions were 39, 33, and 23% at 7 dpi and 66, 34, and 35% at 14 dpi for RKN, WS, and RKN_WS, respectively (Figure 2B and Supplementary Table 4).

### Differentially Expressed Genes Validation by Reverse Transcription-PCR

Ten genes were selected within the up and downregulated DEGs and analyzed by RT-qPCR at 7 and 14 days to evaluate the quality of gene expression profiles by RNA-Seq. The expression pattern of *Polyphenol oxidase* (Solyc08g074630.2.1), *1-aminocyclopropane-1-carboxylate oxidase* (aco5) (Solyc07g026650.3.1), *Heat stress transcription factor B-2b* (Solyc08g080540.3.1), *17.4 kDa class III heat shock protein* (Solyc03g123540.3.1), *Oleosin* (Solyc03g112440.1.1), *COBRA-like protein* (Solyc03g114900.3.1), *LEXYL2* (Solyc01g104950.4.1), *Fasciclin-like arabinogalactan protein 2* (Solyc07g045440.1.1), *Xyloglucan endotransglucosylase/hydrolase 6* (Solyc11g066270.3.1), and *Auxin response factor 9B* (Solyc08g008380.4.1) are shown in Supplementary Figures 1, 2. The expression trends obtained by RT-qPCR were consistent with the results observed by RNA-Seq, confirming the reliability of the DEGs data produced.

### Responses of Abscisic Acid-Related Genes in Water-Stressed Roots and Galls

Dehydration induces transcriptional regulation of abscisic acid (ABA) biosynthetic genes (Xiong and Zhu, 2003) and plant responses to this hormone (Fujita et al., 2011). In order to verify the efficacy of the applied water stress, the expression level of genes associated with ABA synthesis and response were monitored (Supplementary Figure 4). Water stress significantly affected the expression of *NCED1* which encodes the 9-*cis*-epoxycarotenoid-dioxygenase (EC 1.13.11.51). This gene was downregulated in WS roots and RKN_WS galls with respect to C roots. Likewise, *AO3*, which encodes for an aldehyde oxidase (EC 1.2.3.1) key enzyme in the ABA biosynthesis, was downregulated in both WS roots and RKN_WS galls. These results could be peculiar of a late response as the samplings were carried out several days after the imposition of water stress. Indeed, several studies have shown that, at the onset of water stress, ABA increases largely within a few hours, then drops rapidly to a pre-stress value (Cohen et al., 1999). On the other hand, ABA-responsive genes were upregulated upon water-stress. The tomato dehydrin gene *TAS14* is clearly induced by water stress in WS roots and in RKN_WS galls, whereas in RKN galls, the expression was slightly lower than in control roots. Solyc03g116390.2.1, a coding for a late embryogenesis abundant (LEA) protein, was already shown to be inducible by water stress (Gong et al., 2010) along with other two LEA genes (Solyc12g098900.1.1, Solyc10g078770.1.1) that were significantly upregulated in WS and RKN_WS conditions at both times of observation. The upregulation of these marker genes confirmed the effect of the imposed stress.

### Effect of Water Stress and Nematode Infection on Tomato Transcriptome

**FIGURE 3:**
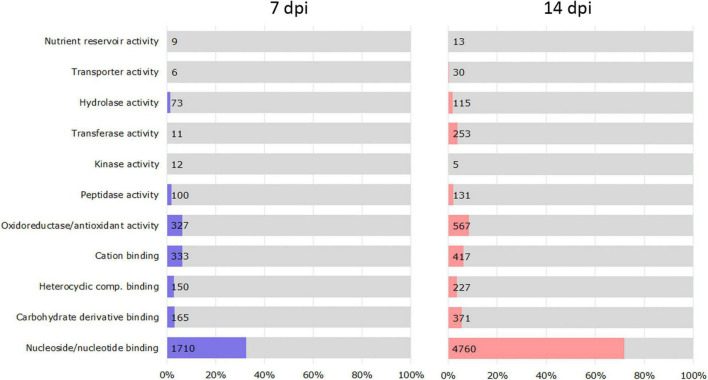
Classification of DEGs based on enriched molecular function in RKN_WS at 7 and 14 dpi. The vertical axis indicates molecular function information, and the horizontal axis indicates the number and percentages of genes enriched in each category. Values were calculated respect to total RKN_WS DEGs [Fold change ≥ 2 or ≤–2, *p*-value ≤ 0.05 and transcripts per million (TPM) ≥ 5].

We examined DEGs (5,254 at 7 dpi and 6,623 at 14 dpi) in the RKN_WS condition to investigate the effect of combined stress on plants (Supplementary Tables 5, 6). A GO enrichment analysis showed that the functional categories significantly enriched in each time sampling were as follows: binding of nucleosides/nucleotides, carbohydrates, heterocyclic compounds, and cations; and oxidoreductase/antioxidant, peptidase, kinase, transferase, hydrolase, transporter, and nutrient reservoir activities (Figure 3). Most of them were downregulated both at 7 and 14 dpi (Table 2).

**TABLE 2:** Gene Ontology (GO) analysis summary: molecular functions were enriched (*p*-value ≤ 0.05) for differentially expressed genes (DEGs) during water stress and *M. incognita* infection (RKN_WS vs. C) at 7 and 14 dpi.

Molecular function	Term	Description	7 dpi	14 dpi
Nucleoside/nucleotide binding	GO:0000166	Nucleotide binding		−
	GO:0001882	Nucleoside binding	−	±
	GO:0017076	Purine nucleotide binding	−	±
	GO:0001883	Purine nucleoside binding	−	±
	GO:0030554	Adenyl nucleotide binding	−	±
	GO:0032549	Ribonucleoside binding	−	±
	GO:0032550	Purine ribonucleoside binding	−	±
	GO:0032553	Ribonucleotide binding	−	±
	GO:0032555	Purine ribonucleotide binding	−	±
	GO:0032559	Adenyl ribonucleotide binding	−	±
	GO:0035639	Purine ribonucleoside triphosphate binding	−	±
	GO:1901265	Nucleoside phosphate binding		−
	GO:0005524	ATP binding	−	±
	GO:0097367	Carbohydrate derivative binding	−	−
Heterocyclic comp. binding	GO:0020037	Heme binding	−	−
	GO:0046906	Tetrapyrrole binding	−	−
Ion binding	GO:0046914	Transition metal ion binding	±	±
	GO:0005506	Iron ion binding	−	−
	GO:0008270	Zinc ion binding	+	+
	GO:0005509	Calcium ion binding	−	−
Oxidoreductase/antioxidant activity	GO:0016209	Antioxidant activity	−	−
	GO:0016491	Oxidoreductase activity	−	−
	GO:0016684	Oxidoreductase act., acting on peroxide as acceptor	−	−
	GO:0016701	Oxidoreductase act., acting on single donors with incorporation of molecular oxygen	−	−
	GO:0004601	Peroxidase activity	−	−
	GO:0015035	Protein disulfide oxidoreductase activity		−
	GO:0004097	Catechol oxidase activity	−	
Peptidase activity	GO:0008236	Serine−type peptidase activity	−	−
	GO:0004252	Serine−type endopeptidase activity	−	
	GO:0070008	Serine−type exopeptidase activity	−	−
	GO:0004185	Serine-type carboxypeptidase activity		−
	GO:0070001	Aspartic-type peptidase activity	−	−
	GO:0004190	Aspartic-type endopeptidase activity	−	−
	GO:0004866	Endopeptidase inhibitor activity		−
Kinase activity	GO:0008443	Phosphofructokinase activity	−	
	GO:0003872	6−phosphofructokinase activity	−	
	GO:0000155	Phosphorelay sensor kinase activity		+
Transferase activity	GO:0046912	Transferase act., transferring acyl groups, acyl groups converted into alkyl on transfer		−
	GO:0016762	Xyloglucan:xyloglucosyl transferase activity	−	−
Hydrolase activity	GO:0004553	Hydrolase act., hydrolyzing *O*-glycosyl compounds	−	−
	GO:0017171	Serine hydrolase activity	−	−
Transporter activity	GO:0090484	Drug transporter activity		−
	GO:0015238	Drug transmembrane transporter activity	−	−
	GO:0045735	Nutrient reservoir activity	−	−

**FIGURE 4:**
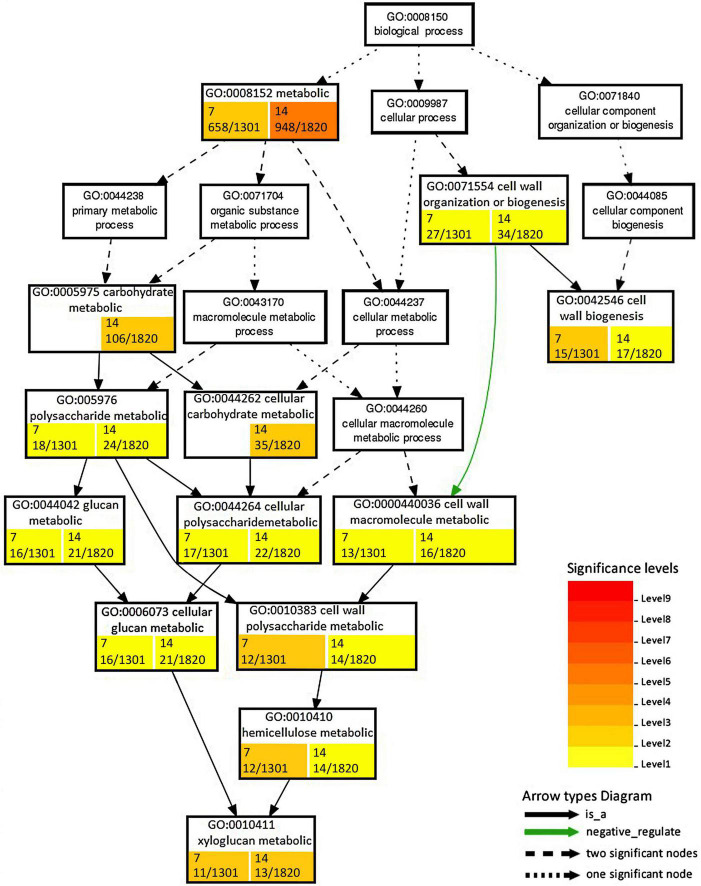
Enrichment analysis of RKN_WS DEGs using Gene Ontology (GO) terms in the “Biological process” related to cell wall. Number of DEGs ascribed to each GO category with respect to total DEGs considered in the analysis are shown in colored box at 7 and 14 dpi. Color is proportional to the level of significance, as indicated by the scale in the legend.

Changes due to the persistence of the stress conditions were found for functions ascribed to nucleoside/nucleotide binding and phosphorelay sensor kinase activity, which showed upregulation after 14 dpi. DEGs that downregulated at 14 dpi included Ca ion binding, protein disulfide oxidoreductase activity, serine-type carboxypeptidase activity, endopeptidase inhibitor activity, transferase activity transferring acyl groups, and drug transporter activity (Table 2). Among enriched biological processes, we found 34 and 53 downregulated and 8 and 49 upregulated GO terms at 7 and 14 dpi, respectively (Supplementary Table 7). The processes associated with cell wall organization or biogenesis were mostly downregulated and showed differences in enriched GO terms and number of DEGs (Figure 4).

**FIGURE 5:**
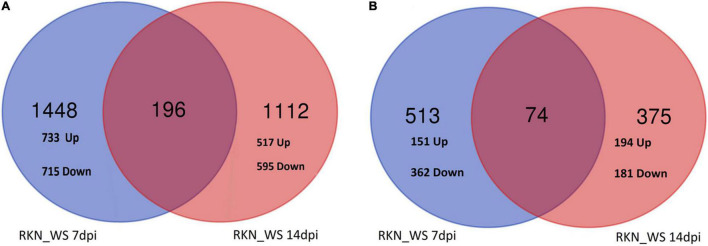
Venn analysis of RKN_WS DEGs exclusive **(A)** and in common with RKN **(B)**, along the two sampling times.

Focusing on DEGs exclusive for RKN_WS treatment were previously shown in Figure 2B, i.e., 1,644 and 1,308 at 7 and 14 dpi, respectively, wherein 733 and 517 genes were upregulated while 715 and 595 genes were specifically downregulated in 7- and 14-days old galls, respectively (Figure 5A).

The analysis indicated that 196 genes, with around 58% upregulated and 42% downregulated, were common to both times of observation (Figure 5A). Interestingly, three genes (Solyc03g123860.4.1, Receptor-like protein kinase; Solyc04g076300.4.1, Mechanosensitive ion channel protein 2, chloroplastic; Solyc02g078760.1.1, hypothetical protein) were upregulated at 7 dpi but downregulated at 14 dpi. Four genes (Solyc06g070900.3.1, TCP transcription factor 17; Solyc12g087860.3.1, RING/U-box superfamily protein; Solyc05g006340.3.1, Transducin/WD40 repeat-like superfamily protein; Solyc06g072690.3.1, Mitotic-spindle organizing protein 1B) were downregulated at 7 dpi, while upregulated at 14 dpi.

Insights into DEGs shared between RKN_WS and RKN galls, shown in Figure 2B, i.e., 587 and 449 at 7 and 14 dpi, respectively, revealed that 74 transcripts were common for both times, whereas 513 and 375 were exclusive at 7 at 14 dpi, respectively (Figure 5B). Among the 513 DEGs, 29% and 71% were, respectively, up and downregulated. The analysis evidenced that water stress changed completely the expression of 19 (from down to up) and 15 (from up to down) genes (Supplementary Table 8). Some of them were involved in signaling receptor, oxidoreductase, transporter, and transcription factor activities. Particularly, the transcription of Solyc04g072070.3.1, the encoding for a WRKY_transcription_factor_55, and upregulation in nematode parasitized roots were inhibited (from 4.4 to −5.18-fold) in galls under water stress (Supplementary Table 8). A further regulatory element markedly affected by water stress was Solyc06g053610.3.1 and the encoding for a R2R3MYB_transcription_factor 14, which changed its expression from down in RKN to up in RKN_WS (−3.5 to 2.6-fold) at 7 dpi. Among the 375 DEGs at 14 dpi, 52 and 48% were, respectively, up and downregulated. Three of them changed from down to up and one from up to down under water stress (Supplementary Table 8). Overall, the time of water deprivation affected the intensity of expression without causing changes in gene trends (Supplementary Table 8).

### Water Stress Modulation of Genes Related to Cell Wall Metabolism in Nematode Feeding Site

Due to the morphology changes observed in nematode FS upon water stress and the GO biological process enrichment, we focalized on genes involved in cell wall biosynthesis, degradation, re-modeling, and extension. Response to water stress was firstly examined in galls of water-stressed plants (RKN_WS) in comparison with the normally watered and uninfected control roots (Supplementary Tables 9, 10). Based on this analysis, 246 (63 upregulated and 183 downregulated) and 288 (55 upregulated and 233 downregulated) DEGs were found in 7- and 14-day-old RKN_WS galls, respectively (Supplementary Table 11). Among them, 181 were present at both infection times. Most DEGs were downregulated with a small number of upregulated genes (Supplementary Table 12). Moreover, only 7 genes showed an opposite regulation between 7- and 14-day-old RKN_WS galls. Four genes, Solyc06g009190.4.1 (Pectinesterase), Solyc11g071470.1.1 (Transferase), Solyc01g080010.2.1 (Xyloglucan endoglucanase inhibitor), and Solyc06g060970.2.1 (Expansin-like B1) were upregulated at 7 dpi but downregulated at 14 dpi. Three genes, Solyc06g060170.3.1 (Pectin lyase-like superfamily protein), Solyc12g008530.2.1 (Pectinesterase), and Solyc06g062580.3.1 (Beta-galactosidase), were downregulated at 7 dpi but upregulated at 14 dpi (Supplementary Tables 9, 10).

Seventy-one out of the 181 DEGs found in both 7- and 14-day-old RKN_WS galls were also present in RKN galls both at 7 and 14 dpi (Table 3) with the same trend, although with different intensity.

**TABLE 3:** DEGs related to the cell wall in common between galls from treatments RKN_WS and RKN at 7 and 14 dpi.

Transcript ID	RKN_WS		RKN		Description
			
	7	14	7	14	
Solyc11g007970.2.1	−1503.76	−4575.84	−12.14	−77.36	4-coumarate–CoA ligase-like 5
Solyc08g068190.3.1	−36.65	−33.43	−8.84	−1697.71	Aldehyde dehydrogenase
Solyc12g007030.3.1	−3.49	−5.32	−2.08	−3.52	Aldehyde dehydrogenase
Solyc02g089170.4.1	−14.58	−139.15	−755.63	−580.39	Alpha−1, 4-glucan-protein synthase
Solyc02g065740.3.1	−22.11	−1200.09	−1753.08	−20.71	Alpha-1, 4-glucan-protein synthase
Solyc07g053640.1.1	2.69	3.67	4.69	2.57	Arabinogalactan-protein
Solyc02g078950.4.1	−8.78	−2936.24	−17.34	−9.53	Beta-galactosidase
Solyc03g019890.3.1	3.3	2.74	4.8	2.63	Beta-galactosidase 7
Solyc07g063390.3.1	−4.68	−3.64	−2.62	−2.94	Beta-glucosidase 16
Solyc01g101120.4.1	4.86	4.03	2.7	2.35	Carbohydrate-binding X8 domain
Solyc09g010200.4.1	−56.74	−5256.02	−2.46	−8.65	Casparian strip membrane protein 1
Solyc04g011480.3.1	−1271.02	−410.76	−23.02	−352.85	CASP-like protein
Solyc02g069730.3.1	16.16	4.67	24.92	7.74	CASP-like protein
Solyc04g051270.2.1	−619.38	−338.07	−531.08	−290.68	CASP-like protein
Solyc11g012590.3.1	3.72	12.01	−2.6	3.85	CASP-like protein
Solyc08g082640.2.1	−3.72	−5.13	−14.47	−4.31	Cellulose synthase
Solyc01g059900.4.1	−5472.7	−7306.08	−4687.24	−64.79	Dirigent protein
Solyc02g032030.1.1	−1788.43	−991.41	−6.68	−852.18	Dirigent protein
Solyc08g081780.1.1	−146.45	−2188.99	−14.31	−9.24	Dirigent protein
Solyc10g008900.3.1	−31.18	−1160.22	−965.99	−997.72	Dirigent protein
Solyc02g083980.3.1	−2.47	−2.14	−5.81	−2.87	Endoglucanase
Solyc08g076640.1.1	−33.29	−3189.76	−1739.85	−69.24	Eukaryotic aspartyl protease family protein
Solyc01g079920.3.1	−1096.42	−886.28	−938.83	−761.85	Eukaryotic aspartyl protease family protein
Solyc08g076630.3.1	−9.34	−1078.72	−20.97	−10.53	Eukaryotic aspartyl protease family protein
Solyc05g012730.1.1	−24.07	−106.8	−5.45	−8.29	Exostosin-like
Solyc08g080060.4.1	−14.47	−579.84	−748.84	−498.99	Expansin
Solyc10g084780.3.1	−27.35	−920.38	−1495.87	−792.06	Expansin
Solyc06g051800.3.1	5.35	1.95	10.48	2.96	Expansin 1
Solyc10g086520.2.1	−11.37	−17.84	−5.72	−6.54	Expansin 6
Solyc12g089380.2.1	−70.45	−43.41	−2362.07	−3055.95	Expansin 8
Solyc05g007830.3.1	8.59	5.24	9.76	4.76	Expansin 12
Solyc06g005560.4.1	8.75	9.46	14.28	12.86	Expansin 9
Solyc01g107220.2.1	−3.87	−5.17	−27.75	−9.61	Extensin-2-like
Solyc11g065910.1.1	−79.94	−223.25	−52.11	−101.94	Extensin-2-like
Solyc03g082790.4.1	−11424.49	−12747.98	−9801.77	−70.53	Extensin-like 54
Solyc01g005850.2.1	−6.12	−9.68	−5.14	−30.13	Extensin-like protein Dif54
Solyc01g097680.2.1	−1006.4	−3158.2	−105.17	−20.03	Extensin-like protein Dif10
Solyc02g030220.1.1	−59.08	−103.67	−48.94	−1116.38	Extensin-like protein Dif10
Solyc03g082770.1.1	−30.11	−28.61	−793.28	−687.37	Extensin-like protein Dif10
Solyc12g100080.1.1	−1178.66	−2207.66	−1010.44	−24.33	Extensin-like protein Ext1
Solyc12g100110.1.1	−1571.56	−4183.64	−1346.01	−64.78	Extensin-like protein Ext1
Solyc12g006110.3.1	−551.93	−16555.46	−8.2	−42.99	Fasciclin-like arabinogalactan protein 2
Solyc02g088500.1.1	−3.5	−2.64	−3.74	−2.84	Glycosyltransferase
Solyc12g010200.2.1	−3.17	−3.26	−3.92	−2.05	Hexosyltransferase
Solyc07g005760.3.1	−2.85	−9.09	−3.65	−4.58	Hydroxycinnamoyl CoA quinate transferase
Solyc02g084990.3.1	2.22	2.32	4.76	3.14	Mannan endo-1, 4-beta-mannosidase
Solyc12g013750.2.1	2.04	3.46	2.25	3.51	Mannan endo-1, 4-beta-mannosidase 1
Solyc05g055490.3.1	−294.21	−1593.95	−18.07	−5.29	Monocopper oxidase-like protein SKU5
Solyc06g007960.4.1	−9.39	−95.01	−6.06	−23.13	*O*-methyltransferase
Solyc03g111690.4.1	8.29	11.28	10.17	11.1	Pectate lyase
Solyc12g019440.3.1	−835.8	−564.02	−715.09	−11.69	Pectin acetylesterase
Solyc01g094970.4.1	5.34	2.67	3.58	4.9	Pectin lyase-like superfamily protein
Solyc12g019120.2.1	−912.6	−749.48	−780.44	−644.87	Pectin lyase-like superfamily protein
Solyc05g005040.4.1	−15.47	−13.52	−7.44	−26.71	Pectin lyase-like superfamily protein
Solyc08g014560.3.1	−688.93	−2452.14	−2174.11	−2107.82	Pectin lyase-like superfamily protein
Solyc12g019130.3.1	−1288.08	−640.72	−1103.89	−551.62	Pectin lyase-like superfamily protein
Solyc12g019140.3.1	−225.89	−1854.83	−3320.17	−21.48	Pectin lyase-like superfamily protein
Solyc03g083840.3.1	−110.35	−4658.06	−4732.88	−4007.47	Pectinesterase
Solyc02g014300.2.1	−26.69	−2090.67	−1339.11	−340.42	Pectinesterase
Solyc12g008530.2.1	−3.16	2.07	−7.19	2.9	Pectinesterase
Solyc10g018320.1.1	−302.97	−423.08	−259.08	−364.13	Plant invertase/pectin methylesterase inhibitor superfamily protein
Solyc03g083660.1.1	−340.41	−1016.1	−291.57	−873.43	Plant invertase/pectin methylesterase inhibitor superfamily protein
Solyc03g083710.1.1	−17.35	−48.38	−5.36	−7.51	Plant invertase/pectin methylesterase inhibitor superfamily protein
Solyc05g005540.4.1	−26.85	−15486.03	−4.19	−29.06	Polygalacturonase-1 non-catalytic subunit beta
Solyc12g056960.2.1	−6.22	−54.19	−2.07	−2.59	Putative glucan 1, 3-beta-glucosidase
Solyc04g076660.3.1	4.18	5.29	7.15	2.85	Rhamnogalacturonate lyase family protein
Solyc03g097500.3.1	21.72	2.06	9.06	3.39	Transferase
Solyc11g071470.1.1	4.17	−3.7	3.61	−2.86	Transferase
Solyc01g080010.2.1	21.95	−2.51	9.65	−2.27	Xyloglucan endoglucanase inhibitor
Solyc12g007250.1.1	−529.09	−824.11	−453.18	−708.43	Xyloglucan endotransglucosylase/hydrolase
Solyc11g040140.2.1	−16.18	−3466.93	−2050.1	−2981	Xyloglucan endotransglucosylase/hydrolase

*Values indicate the fold change respect to uninfected and unstressed control plants.*

### Expression of Feeding Site Genes Involved in Cell Wall Biogenesis

Expression of genes related to cell wall metabolism in RKN_WS were compared to that of the other conditions. Analysis showed that most members of the large cellulose synthase (CesA) and the cellulose synthase-like (Csl) enzyme families were downregulated either in unstressed or stressed galls. The only exception was *CesA* Solyc11g005560.3.1 which was upregulated at 7 and 14 dpi with a higher level of expression at 14 dpi and found in both types of FS (Figure 4). In addition, we found that water stress repressed the expression of the *Csl* genes Solyc08g006310.3.1, Solyc09g075550.3.1, and Solyc12g014430.2.1 that were upregulated at 7 dpi in RKN galls. On the other hand, Solyc03g005450.3.1 was induced by water stress in RKN_WS galls at 14 dpi (Supplementary Tables 9, 10).

**FIGURE 6:**
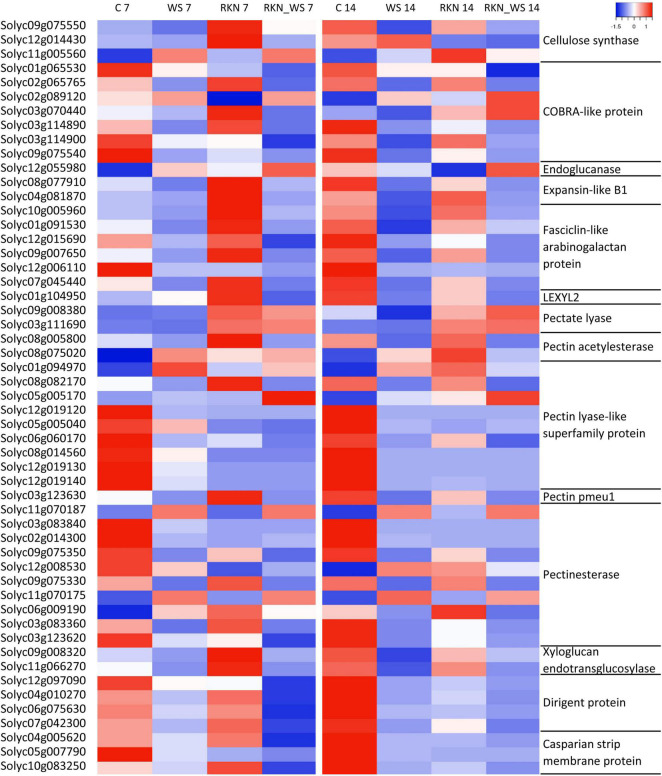
Gene expression patterns of subsets of DEGs associated with cell wall in tomato. The heatmap represents TPM mean values of subsets selected in water stressed roots inoculated with *M. incognita* (RKN_WS) with statistically significant differences (*p*-value ≤ 0.05) for almost one condition. Each row represents a gene, and each column represents an experimental condition at 7 and 14 dpi. Labels on the left and right sides of the heatmap show the Solyc id and annotation, respectively. The color key scale values range from –1 (lowest TPM number) to 1 (highest TPM number).

We also found that fasciclin-like arabinogalactan proteins (FLAs), cell wall structural glycoproteins that mediate cellulose deposition and cell wall development, were differentially expressed in all stress conditions evaluated. In RKN_WS galls, we found 10 DEGs coding for FLAs 1, 2, 4, 9, 11, and 12. Water stress suppressed their expression, and the negative effect was, in some cases, enhanced by the nematode presence (Figure 6). A striking example is Solyc12g006110.3.1, coding for FLA 2, which was downregulated by 551.9 times in RKN_WS with respect to control, and around 55 times with respect to water-stressed and uninfected roots (WS). Likewise, comparison of the expression trends between RKN and RKN_WS galls highlighted an increased downregulation during the double stress condition at both 7 and 14 dpi. In particular, Solyc07g045440.1.1 showed an opposite expression trend at 7 dpi (from 2.02-fold upregulation in RKN to −3.64-fold downregulation in RKN_WS compared to control) (Figure 6 and Supplementary Table 1). Solyc07g045440.1.1 was selected for RT-qPCR validation, and the results confirmed RNA-seq data for all conditions and tested times (Supplementary Figure 2B). In addition, Solyc10g005960.1.1, Solyc01g091530.4.1, and Solyc09g007650.3.1, which were upregulated in RKN galls at 7 dpi, were significantly repressed by water stress (Figure 6 and Supplementary Table 1).

Likewise, water stressed galls showed differential expression of regulatory elements. COBRA proteins play an important role in the cellular architecture and root growth, acting through the organization of cellulose microfibrils orientation. Five *COBRA-like* genes (Solyc01g065530.3.1, Solyc02g065765.1.1, Solyc03g114890.4.1, Solyc03g114900.3.1, and Solyc09g075540.1.1) were found downregulated in RKN_WS galls both at 7 and 14 dpi (Figure 6 and Supplementary Tables 9, 10). Comparing RKN_WS with RKN galls, the major change was observed for Solyc03g114890.4.1, whose expression was sixfold more inhibited upon water stress at 14 dpi. Moreover, two transcripts encoding COBRA proteins, Solyc02g089120.4.1 and Solyc03g070440.4.1, were upregulated in RKN_WS galls at 14 dpi. In addition, their level of expression was higher than in RKN galls (Figure 6 and Supplementary Table 1). Solyc03g114900.3.1 was validated by RT-qPCR and results were consistent with RNA-Seq data for all conditions and tested times (Supplementary Figure 2C).

Among the genes involved in phenylpropanoid pathway, we found that *4-coumarate-CoA ligase* (4CL), *hydroxycinnamoyl CoA quinate transferase* (HCT), *Caffeoyl-CoA O-methyltransferase* (CCoAMT), *Cinnamyl alcohol dehydrogenase* (CAD), and *cinnamoyl-CoA reductase 2* (CCR2) were downregulated at 7 and 14 dpi in galls of stressed and normally watered plants (Supplementary Tables 9, 10). We also found changes in expression of genes involved in the regulation of lignification. Eight dirigent proteins genes (*DIR*), ensuring the stereoselectivity of coniferyl alcohol dimerization and likely mediating the free radical coupling of monolignol plant phenols to yield lignans and lignins, were differentially expressed in response to both biotic and abiotic stress (Supplementary Tables 9, 10). Solyc04g010270.1.1, Solyc06g075630.4.1, Solyc07g042300.1.1, and Solyc12g097090.3.1 were downregulated at both times of observation when compared to C and RKN. In particular, Solyc12g097090.3.1 and Solyc04g010270.1.1 were, respectively, 250- and 20-fold more downregulated with respect to RKN at 7 dpi (Figure 6 and Supplementary Table 1). In addition, Solyc06g054320.1.1 and Solyc10g084440.3.1 were upregulated at 14 dpi (Supplementary Tables 9, 10). DIR proteins interact in some way with Casparian strip domain proteins (CASPs). The latter constitutes a protein complex directing transport across the plasma membrane and recruiting proteins required for Casparian strip (CS) formation and lignification. Our data showed that three CASPs (Solyc04g005620.3.1, Solyc05g007790.3.1, and Solyc10g083250.2.1) were downregulated by nematode infection, particularly at 14 dpi, and that combined stresses enhanced their downregulation. By contrast, we found five CASP-like proteins that were upregulated in RKN_WS galls at both times of observation (Figure 6 and Supplementary Tables 9, 10).

### Expression of Genes Involved in Cell Wall Remodeling

Water stress downregulated most of the gene coding for endo-β-1,4-glucanases (EGase, EC 3.2.1.4) and involved in many processes requiring cell wall modifications. Despite this, it triggered a higher expression of an *EGase* (Solyc12g055980.1.1) in 7-day-old RKN_WS galls (Figure 4). In addition, we found that *EGase cel 8* (Solyc08g082250.3.1), known to be induced by nematode infection in syncytia and giant cells in several plant-nematode interactions (Sobczak et al., 2011), was over-expressed at 7 dpi during the *M. incognita*-tomato interaction in both unstressed and stressed galls, with an expression level higher in presence of nematode only (Supplementary Tables 9, 10).

Analysis of gene expression in RKN_WS galls also showed changes in abundance of several pectin esterase coding genes (Supplementary Tables 9, 10). In particular, Solyc11g070187.1.1 and Solyc11g070175.1.1 were upregulated both at 7 and 14 dpi, whereas Solyc03g083840.3.1, Solyc02g014300.2.1, Solyc09g075350.4.1, Solyc09g075330.4.1, Solyc03g083360.3.1, and Solyc03g123620.4.1 were downregulated at 7 and 14 dpi. Only Solyc06g009190.4.1 was upregulated at 7 dpi and downregulated at 14 dpi, whereas Solyc12g008530.2.1 (downregulated at 7 dpi) was upregulated at 14 dpi (Figure 6 and Supplementary Tables 9, 10). In general, the expression patterns of pectin esterase genes observed for RKN_WS galls were similar to those of RKN galls (Table 3).

Several genes belonging to the pectin lyase-like superfamily, whose members hydrolyze methylated pectin, showed differential expression in RKN_WS galls (Supplementary Tables 9, 10). In particular, two genes (Solyc01g094970.4.1 and Solyc05g005170.4.1) were upregulated at both 7 and 14 dpi, and seven (Solyc08g082170.4.1, Solyc12g019120.2.1, Solyc05g005040.4.1, Solyc12g019140.3.1, Solyc08g014560.3.1, Solyc06g060170.3.1, and Solyc12g019130.3.1) were downregulated at both 7 and 14 dpi (Figure 6 and Supplementary Tables 9, 10). Expression of the aforementioned genes showed the same trend in RKN galls, except for Solyc08g082170.4.1 which was repressed by water stress in RKN_WS galls at 7 dpi compared to galls of normally watered plants (Figure 6 and Supplementary Table 1).

Polygalacturonases and pectate lyases are unable to degrade methylated pectin as they can only work once the pectin is demethylated or deacetylated by pectin methylesterases or acetylesterases. In water-stressed galls, these genes were downregulated at both times of observation except for the pectin acetyl esterase gene Solyc08g075020.3.1 which was upregulated at 7 dpi (Figure 6). Interestingly, a *pectin acetylesterase* (Solyc08g005800.4.1) and the *pectin methylesterase pmeu1* (Solyc03g123630.4.1), which were downregulated in response to water stress, were upregulated in RKN galls at 7 dpi (Figure 6 and Supplementary Table 1).

Moreover, genes coding for polygalacturonases were downregulated at both 7 and 14 dpi in RKN_WS and in RKN galls. Among genes coding for pectate lyases, two (Solyc09g008380.3.1 and Solyc03g111690.4.1) were upregulated in RKN_WS galls at 7 dpi, and Solyc03g111690.4.1 was also up-regulated at 14 dpi (Figure 6). Another three genes coding for pectate lyase were downregulated in 14-day-old RKN_WS galls (Supplementary Tables 9, 10). Our results showed that the expression of a *xyloglucan endotransglucosylase-hydrolase 6* (Solyc11g066270.3.1) was affected by water stress as the gene, upregulated in RKN galls, and was downregulated in RKN_WS at 7 dpi (Figure 6 and Supplementary Table 1). Likewise, another gene coding for a xyloglucan endotransglucosylase-hydrolase (Solyc09g008320.4.1), which was up-regulated in RKN galls at 7 dpi, was down-regulated in response to water stress at both 7 and 14 dpi (Figure 6 and Supplementary Table 1).

Plant cell wall hemicellulose components, arabinoxylans and xylans, are subject to modification during plant growth and development. We found that *LEXYL2* (Solyc01g104950.4.1) coding for a α-l-arabinofuranosidase, belonging to glycoside hydrolase (GH) family 3 with both α-L arabinofuranosidase and β-xylosidase activities, was downregulated at 14 dpi in RKN and RKN_WS galls. By contrast, at 7 dpi it was upregulated in RKN and downregulated in RKN_WS galls, suggesting a role of LEXYL2 in the early stage of nematode FS development which was negatively affected by water stress (Figure 6 and Supplementary Table 1). The expression of this gene was validated by RT-qPCR, and values were significant (*p* < 0.05) compared with control and trends were consistent with RNA-Seq data (Supplementary Figure 2A).

Also, the expression analysis of members of the expansin families revealed five downregulated and three upregulated genes at both 7 and 14 dpi in RKN_WS galls. *Expansin 9* (Solyc06g005560.4.1) and *expansin 12* (Solyc05g007830.3.1) were specifically activated, whereas *expansin 6* (Solyc10g086520.2.1) and *expansin 8* (Solyc12g089380.2.1) were repressed in water-stressed galls compared to control plants (Table 3). These genes showed the same expression trend in RKN galls. By contrast, expansin A11 (Solyc04g081870.4.1) and expansin-like B1 (Solyc08g077910.3.1) showed opposite expression patterns in the different type of FS. Particularly, upregulated in 7 dpi RKN galls and repressed by water stress in 7- and 14-day-old RKN_WS galls (Figure 6 and Supplementary Table 1). The expression of expansin 1 (Solyc06g051800.3.1) in nematode FS was affected by water stress. Although upregulated compared to C plants, it had a lower level of expression (about twofold less) in comparison to RKN galls at 7 dpi (data not shown).

### Distribution of Cellulose in Feeding Site

**FIGURE 7:**
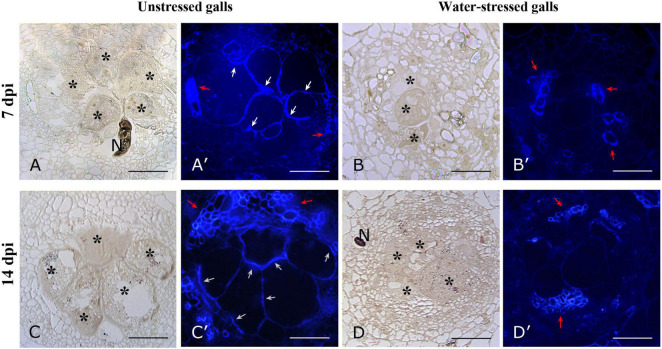
Bright field and fluorescence microscope images of cellulose distribution in cross sections of unstressed (RKN) and water-stressed (RKN_WS) galls stained with Calcofluor-white. Blue fluorescence was present on cellulosic walls of GCs and vessels of unstressed galls at 7 **(A′)** and 14 dpi **(C′)**. No signals were detectable on GCs walls of water-stressed galls both at 7 and 14 dpi **(B′,D′)**, only xylem vessels showed fluorescence. Bright field images of corresponding sections of unstressed **(A,C)** and water-stressed galls **(B,D)**. Asterisks indicate GCs; N, nematode; white arrow, cellulosic cell walls; red arrow, vessels; scale bar, 100 μm.

For the study of cellulose distribution, we used Calcofluor white (Figure 7), which stains cellulose, callose, and other β-glucans (Hughes and McCully, 1975). When excited with UV light, calcofluor white produces a blue fluorescence of cellulosic walls (Kitin et al., 2020). The presence of cellulose was detectable in vessel walls of both RKN and RKN_WS galls (Figures 7A′–D′). By contrast, a stronger signal was only detected along the GC walls in RKN both at 7 and 14 dpi (Figures 7A′,C′).

## Discussion

The current study shows the combination of morphological features and mRNA-Seq data during the tomato-*M. incognita* interaction under a concomitant water stress.

*Meloidogyne* spp. establish a permanent FS by inducing multinucleate hypertrophied GCs which reach their maximum size within 2 weeks. GC wall surface presents ingrowths that interface with xylem cells to increase the plasmalemma surface area and support increased nutrient uptake (Rodiuc et al., 2014). The transfer of nutrients to nematodes involves regulation of water flux into GCs (Baldacci-Cresp et al., 2015). Cell wall thickening and loosening is required to allow GC expansion and to provide mechanical support to maintain cell shape in response to high osmotic pressure (Gheysen and Mitchum, 2009). It has been demonstrated that the nematode nutrient acquisition through GCs relies on the formation of *de novo* formed vascular tissues, and that both primary and secondary cell walls are deposited both in GCs and vascular tissues (Kyndt et al., 2013).

In our study, the decreased tomato growth in aboveground plant parts and roots was a clear response to water stress which could be correlated with endogenous ABA accumulation (Sharp and Davies, 1989). Furthermore, a worsened nematode penetration rate was observed, as demonstrated by the drastic reduction in the number of galls at both sampling times. An increase in endogenous ABA at the onset of drought stress (Cohen et al., 1999) might have stimulated resistance to *M. incognita* in tomato. ABA plays a complex role in the plant’s defense response while it promotes resistance in some plant–pathogen interactions, whereas it increases susceptibility in others (Asselbergh et al., 2007; Ton et al., 2009). Reports on the direct influence of ABA on plant responses to nematode attack remain controversial. Experiments with exogenous ABA revealed that ABA plays a negative role in rice defense against the migratory nematode *Hirschmanniella oryzae* in roots (Nahar et al., 2012). Nevertheless, Karimi et al. (1995) reported a lower reproduction of *M. incognita* on potato roots of ABA-treated plants. In our study, the effects of water stress on the FS development at 7 dpi were remarkable. The GCs were smaller and delayed in development, and their walls were thinner compared to those of the GCs in normally watered plants. In time, the GCs in stressed plants looked similar to those in the 14 dpi normally watered ones, although they continued to be smaller. These changes could be orchestrated by ABA which has a role in prevention of cell-wall loosening (Gimeno-Gilles et al., 2009) and/or secondary cell wall formation (Liu et al., 2021; Yu et al., 2021). In this context, we tried to characterize the transcriptomic landscape of nematode FS and its adaptation in response to water stress in 7- and 14-day-old galls. The expression analysis was carried out in whole tomato galls, including GCs, neighboring cells, and vascular tissues, therefore reflecting the changes occurring during the tomato-*M. incognita* interaction.

Enrichment analysis allowed to highlight that the persistence of the water stress condition triggers the modulation of genes involved in specific cellular functions and biological processes. In particular, GO categories, such as binding, kinase and transporter activities, and cell wall related processes, showed different trends along the experimental conditions. Thus, we focused our attention on those transcripts involved in cell wall synthesis and modification during gall formation. The plant cell wall is a complex and dynamic structure with an important role in cell and organ growth and intercellular communication. It also provides mechanical strength to withstand turgor pressure and reacts to different biotic and abiotic stresses to allow proper development and differentiation of plant tissues and organs (Houston et al., 2016).

### Cell Wall Biogenesis in Galls Induced by *M. incognita*

The cell walls consist of primary and secondary walls whose typical components are cellulose, non-cellulosic, and pectic polysaccharides, enzymatic and catalytic proteins, phenolic compounds, and water. A number of studies report that cellulose biosynthesis can be altered in response to water deficit, as shown by the decreased level of cellulose content in several different species (Le Gall et al., 2015). We found that the distribution of cellulose resulted in altered nematode FS of plants subjected to water stress. The fluorescent dye only highlighted cellulose in GC walls of unstressed galls. Moreover, our expression analyses confirmed that genes involved in cellulose biosynthesis had a different expression pattern in response to water stress during the FS development. Generally, water stress repressed the expression of genes belonging to the Csl family. In particular, the dramatic downregulation of *cellulose synthase-like* Solyc07g043390.4.1 triggered by water stress mainly at 14 dpi could be associated to a reduced FS growth. This finding is in agreement with Choe et al. (2021) who reported that downregulation of this gene by tomato yellow leaf curl virus infection caused a stunted growth phenotype in tomato. Moreover, Solyc07g043390.4.1-silenced tomato plants displayed changes in stem anatomy with cell size reduction and inhibition of secondary xylem (Choe et al., 2021). This suggests that this *cellulose synthase-like gene* plays a critical role in cell wall metabolism in different tomato tissues.

Among many genes involved in cellulose synthesis and deposition, members of the COBRA multigene superfamily act in the assembly of cellulose micro-fibrils at least by partially modulating cellulose crystallinity (Liu et al., 2013) and by regulating the orientation of cell expansion (Schindelman et al., 2001). COBRA encodes an extracellular glycosylphosphatidylinositol (GPI)- anchored protein which is stably associated with the plasma membrane by its GPI-anchor and is required for cell wall synthesis and morphogenesis (Gillmor et al., 2005). Each COBRA member is active in diverse biological processes during cell expansion and cell wall biosynthesis (Roudier et al., 2005). This is also confirmed in our data, with some genes downregulated and others upregulated during the root-*M. incognita* interaction. Interestingly, water stress intensified these effects. Thus, the differential regulation observed for COBRA genes in stressed gall suggests that they are specifically responsive to water stress and could be directly involved in modifications of the cell wall through changes in the orientation of cellulose micro-fibrils.

Furthermore, in our transcriptome analysis, three genes (Solyc10g005960.1.1, Solyc01g091530.4.1, and Solyc09g007650.3.1) coding for Fasciclin-like arabinogalactan proteins (FLAs) were specifically upregulated during the tomato-*M. incognita* interaction. FLAs are a subclass of arabinogalactan proteins (AGPs) that play a role in secondary cell wall assembly and may physically interact with the cellulose synthase complex. They are characterized by containing one or two FAS1 (fasciclin-like) domains and at least one AGP module, and often contain a C-terminal GPI anchor signal peptide. Plants may express some FLAs with GPI for maintaining the integrity of the plasma membrane and others that are not GPI-anchored for mediating cell expansion (Johnson et al., 2003). According to a recent study, GPI-anchored function might also be involved in host–pathogen interactions (Wu et al., 2020). All tomato upregulated FLAs showed a potential C-terminal GPI-modification site (predicted by big-PI Plant Predictor^11^). In unstressed FS, the accumulation of fasciclins could promote cell wall expansion. This latter, in turn, can provide a greater surface area for water absorption and nutrient uptake, thus ensuring nematode development. Moreover, FLAs are also involved in signaling as they can control the transcriptional program for cell wall formation (Huang et al., 2013). In this regard, knock down of FLA6 in *Populus* resulted in a decrease of lignin and cellulose content in the xylem and in a downregulation of some xylem-specific genes associated with cellulose and lignin biosynthesis (Wang et al., 2015). Under water stress, the expression levels of tomato FLAs appeared dramatically decreased. Therefore, alterations in FLAs’ transcript abundance upon water stress could induce changes in expression of other genes involved in cell wall metabolism and presumably affect its chemical composition. Similar evidence was found for two AGP in *Pinus taeda* (No and Loopstra, 2000), which was likely an effect of growth inhibition induced by drought.

Our results also showed a general downregulation of genes involved in monolignol biosynthesis in both unstressed and water-stressed FS. These results were consistent with previous findings observed during *M. incognita*-tomato interaction (Veronico et al., 2018) where repression of some gene functioning in the core of phenylpropanoid pathway and in lignin biosynthesis was found in galls at 7 dpi. Similarly, a global downregulation of phenylpropanoid pathway was reported in rice (Kyndt et al., 2012) and poplar (Baldacci-Cresp et al., 2016) galls and in micro-dissected GCs in both tomato and Arabidopsis (Portillo et al., 2013). The root-knot nematodes likely repress the host defense mechanism to facilitate infection and to establish appropriate gall and GC formation.

Interestingly, our data revealed for the first time an alteration in the expression profiles of most DIR proteins in tomato-*M. incognita* interaction which is likely involved in the production of lignin and lignan (Davin and Lewis, 2000) and therefore participate in plant defense (Paniagua et al., 2017). *DIR* genes were downregulated in response to nematode parasitism and water stress. Considering these results, it is conceivable that DIR proteins could mediate the spatial control of lignin deposition during gall development and in response to water stress.

Dirigent proteins are also required for the correct patterning of lignin deposition in CS (Hosmani et al., 2013), a ring-like cell wall structure in the root endodermis of vascular plants. It is composed of a lignin polymer that is tightly adhered to the plasma membrane and spans the apoplastic space between adjacent endodermal cells. In this way, a barrier that seals the apoplastic pathway in and out of the endodermis is generated and is thus thought to be crucial for selective nutrient uptake, exclusion of pathogens, and many other processes (Enstone et al., 2002). A family of transmembrane proteins called CASPs drives CS formation by accumulating at the appropriate membrane locations (Roppolo et al., 2011). Second-stage endodermis differentiation proceeds with the suberization of entire cell walls where suberin forms a barrier for uptake from the apoplast into the cell interior. In the present study, the expression of many transcript coding for enzymes involved in suberin biosynthesis, including fatty acid hydroxylases, a β-ketoacyl-CoA synthase, fatty acyl-CoA reductases, and long-chain acyl-CoA synthases (data not shown), was activated in tomato upon *M. incognita* parasitism. Meanwhile, expression of genes involved in CS formation was particularly decreased in 14-day-old galls, consistent with what has been found in Arabidopsis by Holbein et al. (2019). Intriguingly, water stress induced a remarkable downregulation of CASP genes from 7 dpi in RKN_WS galls and a concomitant strong activation of genes involved in suberin biosynthesis. These results could reflect changes in root tissues induced by nematodes during various parasitic stages, i.e., endodermis degradation during later stages of FS development and consequent periderm formation (Holbein et al., 2019). However, the sharp downregulation of CASPs at 7 dpi in RKN_WS is a confirmation of how the lignification process is altered by water stress. Therefore, we can speculate that defects in CS formation with simultaneous loss of nutrients could impair nematode development as supported by microscopic observations of smaller FS. In contrast, we found five CASP-like proteins exclusively upregulated in galls of water stressed plants, mainly at 7 dpi. Recent studies revealed that this endodermal barrier can play a central role in mediating signaling to control growth and to respond to external environment (Dinneny, 2014; Vermeer et al., 2014). Therefore, our findings offer cues to further explore the distinguished role of the CS protein family genes in growth, development, and environmental challenges.

### Cell Wall Remodeling in Galls Induced by *M. incognita*

Cell growth and expansion of nematode FS are mediated by the upregulation of gene encoding proteins that promote wall loosening, such as expansins, pectate lyases, pectin methylesterases, and glucanases (Griesser and Grundler, 2008; Wieczorek et al., 2014; Shukla et al., 2018). Plant expansins are also implicated in responses to many abiotic stresses, such as drought, salinity, cold, heat, and oxidative stress (Marowa et al., 2016), indicating that these proteins constitute a common component in the response of plants to stress. They act by weakening the hydrogen bonds between cell wall polysaccharides (McQueen-Mason and Cosgrove, 1994). Our data highlighted the involvement of expansins in the processes that require the expansion and degradation of cell walls. Most gene coding for these proteins were differentially expressed compared to control plants both in unstressed and stressed galls, but mainly in response to nematode parasitism. However, water stress particularly repressed expansin A11 (Solyc04g081870.4.1) and expansin-like B1 (Solyc08g077910.3.1), which were over-expressed in unstressed galls. Interestingly, expansin 1 (Solyc06g051800.3.1), although upregulated upon infection, had a lower level of expression at 7 dpi in stressed galls. Arabidopsis EXPA1 (Jammes et al., 2005) and a poplar homolog to the Arabidopsis *EXPA1* (Baldacci-Cresp et al., 2016) were upregulated during gall development, confirming the hypothesis that these enzymes play a role in the establishment of root-knot nematode parasitism as already suggested in tomato for LeEXPA5 (Gal et al., 2006). In agreement with other plant-microbe interactions (Gal et al., 2006; Dermatsev et al., 2010; Abuqamar et al., 2013), our findings suggest that the decreased expression of some expansin and expansin-like genes could reduce cell wall-loosening activity, resulting in stiffer walls that impede the physical penetration and proliferation of *M. incognita* in tomato.

Likewise, water stress also modified the expression profile of genes involved in the degradation and remodeling of cell wall. In particular, their involvement could be associated with the gall development stage. Although several genes showed the same trend of expression between unstressed and stressed galls, some were specifically up or downregulated by water stress, and this behavior underwent a shift during FS development. Several alterations concerned the genes involved in pectin degradation. Pectins form a hydrated gel, allowing polymer slippage during cell wall growth and facilitate elongation. Interestingly, our findings showed that the gene coding for *pectin acetylesterase* (Solyc08g005800.4.1) and for *pectin methylesterase pmeu1* (Solyc03g123630.4.1), upregulated in FS of normally watered plants, were downregulated in response to water stress. PME3 *Arabidopsis thaliana* mutants overexpressing this gene were more susceptible to the cyst nematode *Heterodera schachtii*, while a knockout mutant showed the opposite effect (Hewezi et al., 2008). Therefore, this allows us to speculate their importance for the establishment of nematode parasitism.

The cell wall of giant cells contains high ester pectic homogalacturonan, xyloglucan, and pectic arabinan (Bozbuga et al., 2018) which are responsible for the flexible properties of FS cell walls. Major cell wall polysaccharides arabinan and arabinoxylan, deriving from arabinose, have an important role in cellular attachment (Iwai et al., 2001) and wall flexibility (Moore et al., 2008). A recent study conducted in young tomato fruit, in which cell wall reconstitution actively occurs, reported an upregulation of α*-l-arabinofuranosidase LeXYL2* (Miyohashi et al., 2021). Authors suggested that this enzyme may contribute to the temporal loosening of the rigid structure that is required for fruit enlargement. We found downregulation of *LEXYL2* in RKN_WS with respect to RKN. Likewise, water stress negatively affected expression of two different genes coding for xyloglucan endotransglucosylase-hydrolases which were upregulated in RKN galls. These enzymes, involved in xyloglucan modification and cell expansion, have also been reported to be upregulated in *Populus-M. incognita* interaction (Baldacci-Cresp et al., 2020). Evidently, water stress modifies processes related to polysaccharide polymerization and/or the mechanism involved in the development of FS where cell enlargement is required. This could explain the changes in the morphology of FS upon water stress.

## Conclusion

This investigation provides novel insights on how water stress modulated tomato response during nematode FS development. These data highlighted that water stress particularly affects genes involved in cell wall metabolism which are important for the successful establishment of parasitism. These findings offer interesting cues for a better understanding of the role of differentially modulated genes during the formation of *M. incognita* GCs. These genes could be potential targets for biotechnological strategies aimed at providing new varieties resistant to root-knot nematodes.

## Data Availability Statement

The original contributions presented in the study are publicly available. This data can be found here: National Center for Biotechnology Information (NCBI) BioProject database under accession number PRJNA734743.

## Author Contributions

AC, MTM, LR, and PV conceived and designed the research. PV, LR, EF, and MC performed the molecular biology experiments and data analysis. MTM performed the histopathology and morphological analyses. IP carried out the bioinformatics analyses. LR and PV wrote the manuscript. AC, FD, EF, and MTM provided editorial advice and revised the manuscript. All authors read and approved the final manuscript.

## Conflict of Interest

The authors declare that the research was conducted in the absence of any commercial or financial relationships that could be construed as a potential conflict of interest.

## Publisher’s Note

All claims expressed in this article are solely those of the authors and do not necessarily represent those of their affiliated organizations, or those of the publisher, the editors and the reviewers. Any product that may be evaluated in this article, or claim that may be made by its manufacturer, is not guaranteed or endorsed by the publisher.

## Footnotes

1
http://imagej.nih.gov/ij/

2
http://www.clcbio.com

3
https://solgenomics.net/organism/Solanum_lycopersicum/genome/

4
http://www.heatmapper.ca/expression/

5
http://bioinfo.cau.edu.cn/agriGO/

6
https://solgenomics.net/organism/Solanum_lycopersicum/genome/

7
http://bioinformatics.psb.ugent.be/webtools/Venn/

8
http://geneontology.org

9
https://solcyc.solgenomics.net/pwy-search.shtml

10
https://www.ncbi.nlm.nih.gov/tools/primer-blast/

11
https://mendel.imp.ac.at/gpi/cgi-bin/gpi_pred_plants.cgi
