# Supplementary material for: Water Stress Differentially Modulates the Expression of Tomato Cell Wall Metabolism-Related Genes in Meloidogyne incognita Feeding Sites
Source: Front Plant Sci. 2022 Apr 15;13:817185. doi: 10.3389/fpls.2022.817185 (PMC9051518; doi:10.3389/fpls.2022.817185)
Supplement: Supplementary file 1 [file Data_Sheet_1.zip › Table S2.docx]

Supplementary table 2. Primers used in RT- qPCR and amplicons length

| Gene | Name | Sequence 5' 3' | Genomic location * | Length |
| --- | --- | --- | --- | --- |
| Heat stress transcription factor B-2b | Sl0540F | gatatgagcccacggttgtt | SL4.0ch08:61927253...61927234 | 245 |
|  | Sl0540R | cacgtgttggtcatcaatgc | SL4.0ch08:61927009...61927028 |  |
| Class II heat shock protein Hsp20 | Sl3540F | tcccgtggacattctggata | SL4.0ch03:64823238...64823219 | 270 |
|  | Sl3540R | aacaccccattctcgcattt | SL4.0ch03:64822901...64822920 |  |
| Oleosin | Sl2440F | cccaacggaggctatcaaaa | SL4.0ch03:57272158...57272177 | 233 |
|  | Sl2440R | acgtcaaaaatccggtgaca | SL4.0ch03:57272390...57272371 |  |
| 1-aminocyclopropane-1-carboxylate oxidase (aco5) | Sl6650F | gctcttgtatccctgtcacc | SL4.0ch07:30390221...30390202 | 284 |
|  | Sl6650R | agcaatcctaactttggcctt | SL4.0ch07:30389938...30389958 |  |
| Polyphenol oxidase | Sl4630F | gcaagttcatttctcgtggc | SL4.0ch08:56869983...56870002 | 254 |
|  | Sl4630R | gtgtttcgacgtccttacca | SL4.0ch08:56870236...56870217 |  |
| LEXYL2 | Sl4950F | agatccaacgctctctagca | SL4.0ch01:85579788...85579769 | 219 |
|  | Sl4950R | acacaactcttgaatggggg | SL4.0ch01:85579475...85579494 |  |
| Fasciclin-like arabinogalactan Protein 2 | Sl5440F | ccatttcgctgctactttcg | SL4.0ch07: 58423513...58423494 | 200 |
|  | Sl5440R | tggctacatctgggtgagat | SL4.0ch07:58423314...58423333 |  |
| Xyloglucan endotransglucosylase /hydrolase 6 | Sl6270F | gaacaacaccaaacaagccat | SL4.0ch11:50031115...50031135 | 299 |
|  | Sl6270R | taattcgtccttcctccgttg | SL4.0ch11:50032093...50032073 |  |
| COBRA like protein | Sl4900F | cttgtggccctgcaaagata | SL4.0ch03:59194872...59194891 | 297 |
|  | Sl4900R | tgcattgcagtagtggttca | SL4.0ch03:59195324...59195305 |  |
| Auxin response factor 9B | Sl8380F | actcgcaccaaggttcaaat | SL4.0ch08:2817318...2817338 | 183 |
|  | Sl8380R | ctgaccaaggataatcgccc | SL4.0ch08:2817510...2817490 |  |
| Actin | Act-for | gctttgccgcatgccattct | SL4.0ch11:278214...278195 | 315 |
|  | Act-rev | gatacctgcagcttccatacc | SL4.0ch11:277900...277920 |  |

* Reference *Solanum lycopersicum* Genome version SL4.0
