# Supplementary material for: Water Stress Differentially Modulates the Expression of Tomato Cell Wall Metabolism-Related Genes in Meloidogyne incognita Feeding Sites
Source: Front Plant Sci. 2022 Apr 15;13:817185. doi: 10.3389/fpls.2022.817185 (PMC9051518; doi:10.3389/fpls.2022.817185)
Supplement: Supplementary file 1 [file Data_Sheet_1.zip › Table S3.docx]

Supplementary table 3: RNA-Seq data produced for single replica and mapping values on *Solanum lycopersicum* genome.

|  |  |  |  |  |  |  |  |  |  |  |
| --- | --- | --- | --- | --- | --- | --- | --- | --- | --- | --- |
| Name | acronym | replica | Total reads (million) |  | Reads mapped on SL4.0 Reads (million) % | | replica | Total reads (million) | Reads mapped on SL4.0 Reads (million) % | |
| Control | C | c_1 | 20,49 |  | 19,09 | 93,04 | c14_1 | 20,74 | 19,15 | 92,31 |
| Control | C | c_2 | 20,8 |  | 19,55 | 94,01 | c14_2 | 18,06 | 16,55 | 91,65 |
| M. incognita | RKN | n7_1 | 21,33 |  | 18,07 | 84,71 | n14_1 | 21,26 | 14,77 | 69,46 |
| M. incognita | RKN | n7_2 | 26,05 |  | 19,47 | 74,73 | n14_2 | 22,52 | 15,91 | 70,64 |
| water stress | WS | ws7_1 | 20,06 |  | 18,5 | 92,21 | ws14_1 | 18,13 | 16,54 | 91,26 |
| water stress | WS | ws7_2 | 22,43 |  | 19,89 | 88,7 | ws14_2 | 29 | 29,97 | 93,03 |
| water stress | WS | ws7_3 | 23,37 |  | 21,78 | 93,23 |  |  |  |  |
| M. incognita  +water stress | RKN_WS | n+ws7_1 | 21,61 |  | 18,11 | 83,81 | n+ws14_1 | 25,77 | 22,07 | 85,64 |
| M. incognita +water stress | RKN_WS | n+ws7_2 | 21,39 |  | 18,83 | 88,05 | n+ws14_2 | 18,44 | 15,69 | 85,14 |
| M. incognita +water stress | RKN_WS | n+ws7_3 | 22,74 |  | 19,51 | 85,79 |  |  |  |  |
| **Total** |  |  | **220,27** |  |  |  |  | **173,92** |  |  |
