# Supplementary figures and images for: Water Stress Differentially Modulates the Expression of Tomato Cell Wall Metabolism-Related Genes in Meloidogyne incognita Feeding Sites
Source: Front Plant Sci. 2022 Apr 15;13:817185. doi: 10.3389/fpls.2022.817185 (PMC9051518; doi:10.3389/fpls.2022.817185)

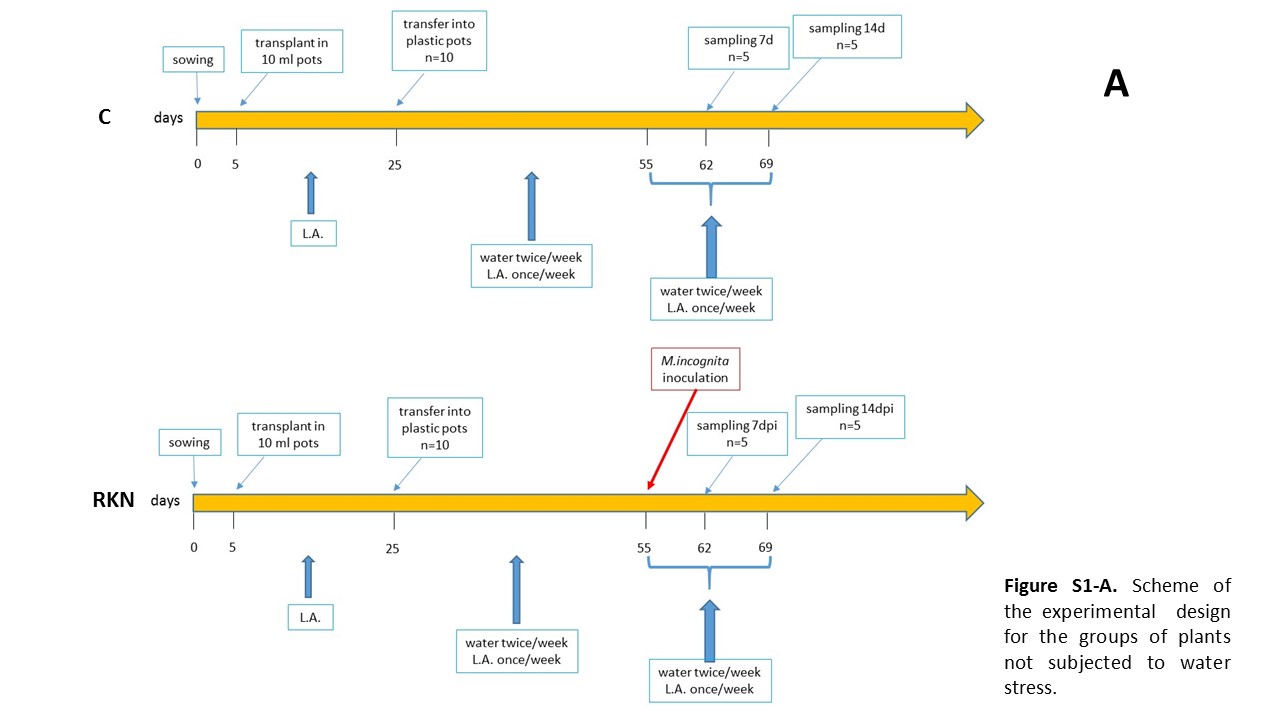

Supplement: Supplementary file 1 [file Data_Sheet_1.zip › Figure S1A.JPEG]

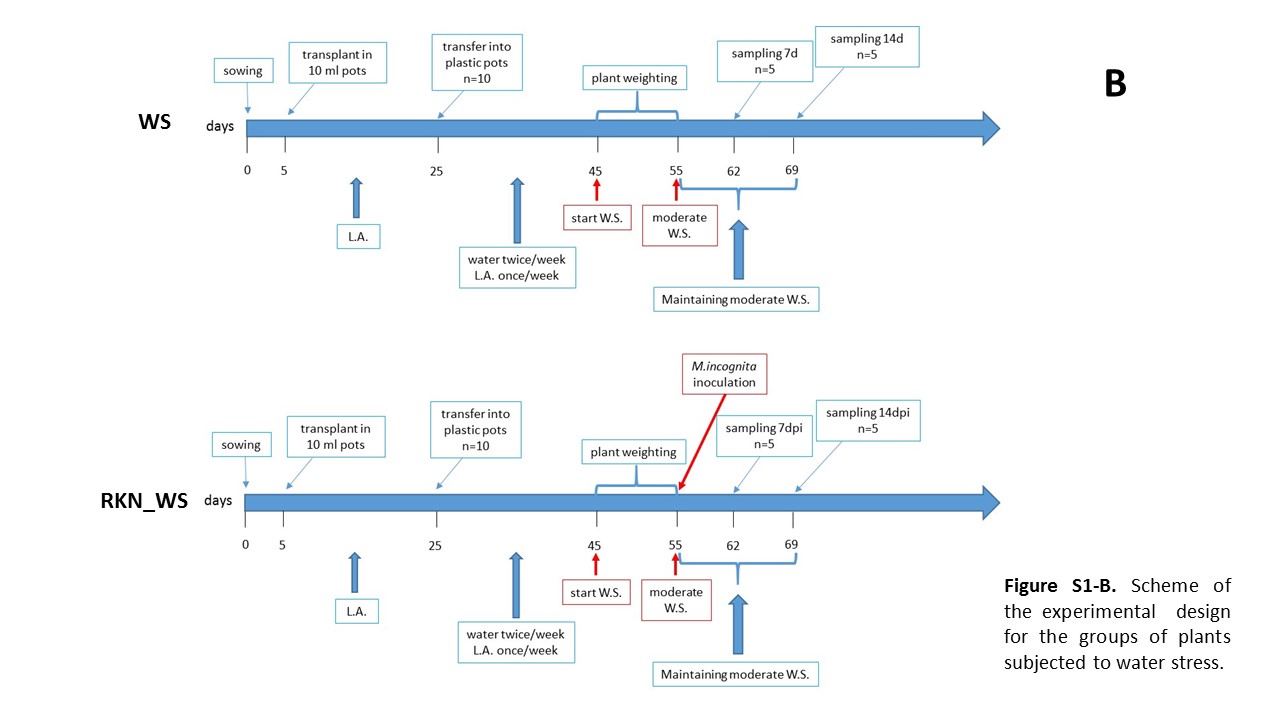

Supplement: Supplementary file 1 [file Data_Sheet_1.zip › Figure S1B.JPEG]

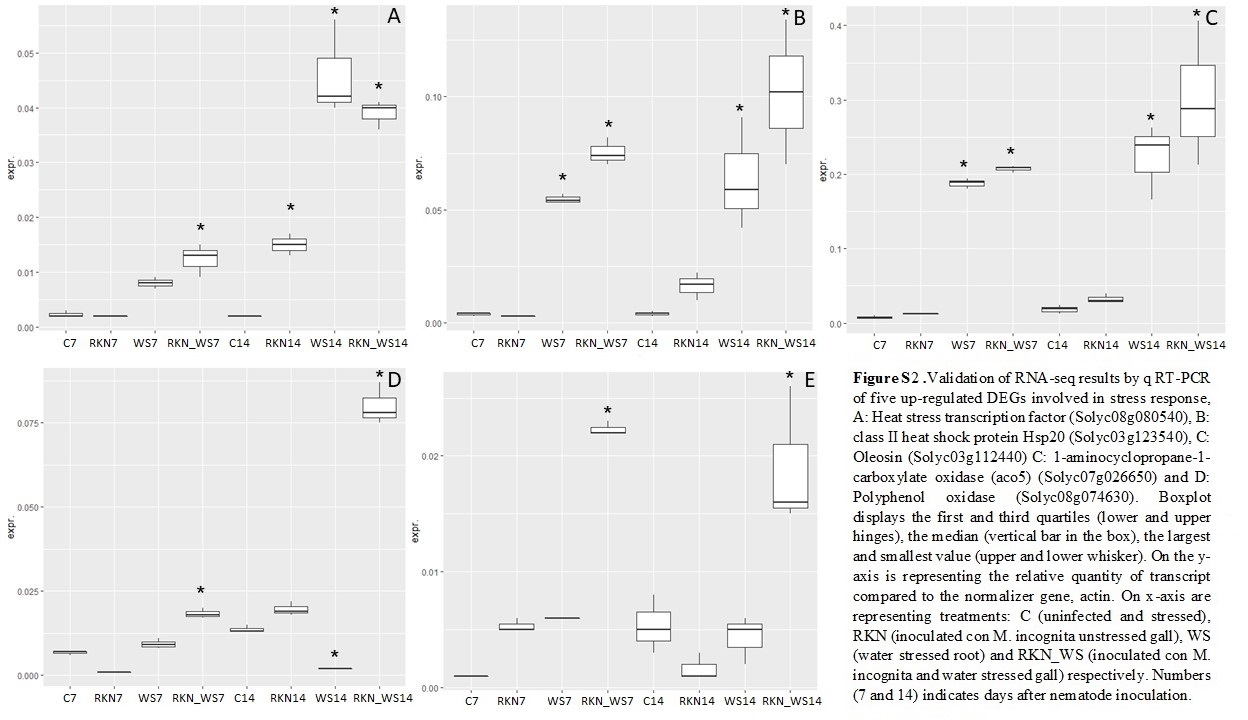

Supplement: Supplementary file 1 [file Data_Sheet_1.zip › Figure S2.JPEG]

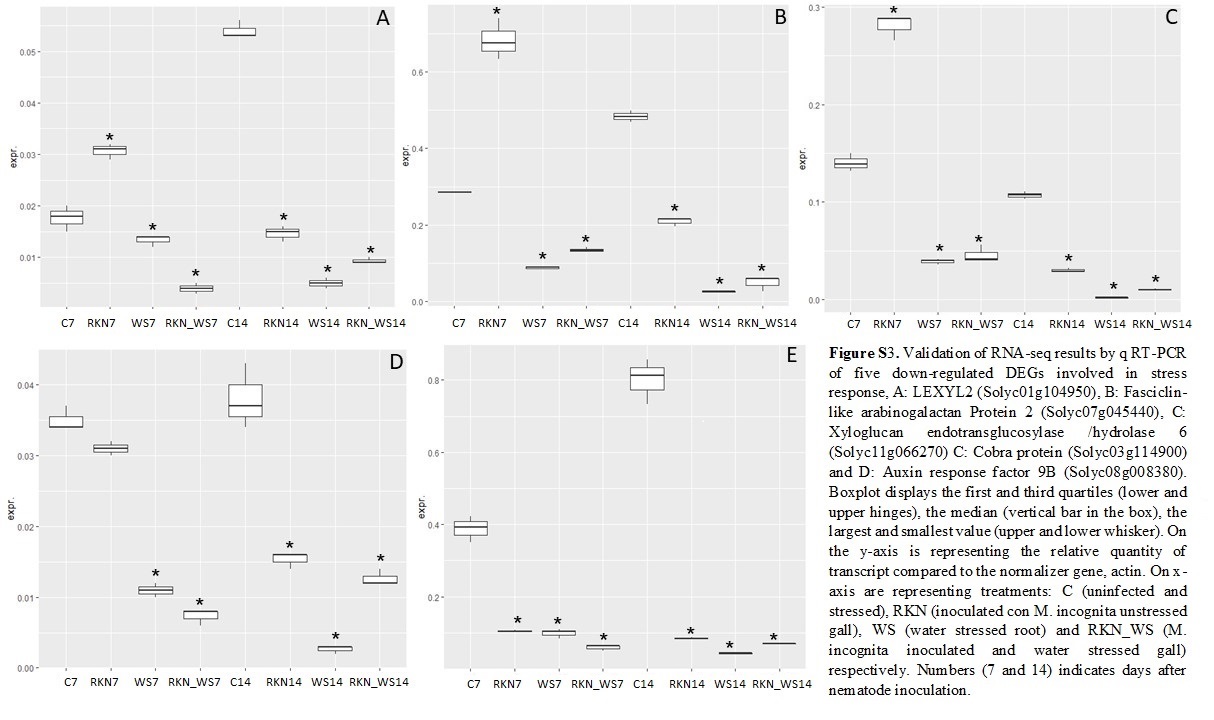

Supplement: Supplementary file 1 [file Data_Sheet_1.zip › Figure S3.JPEG]

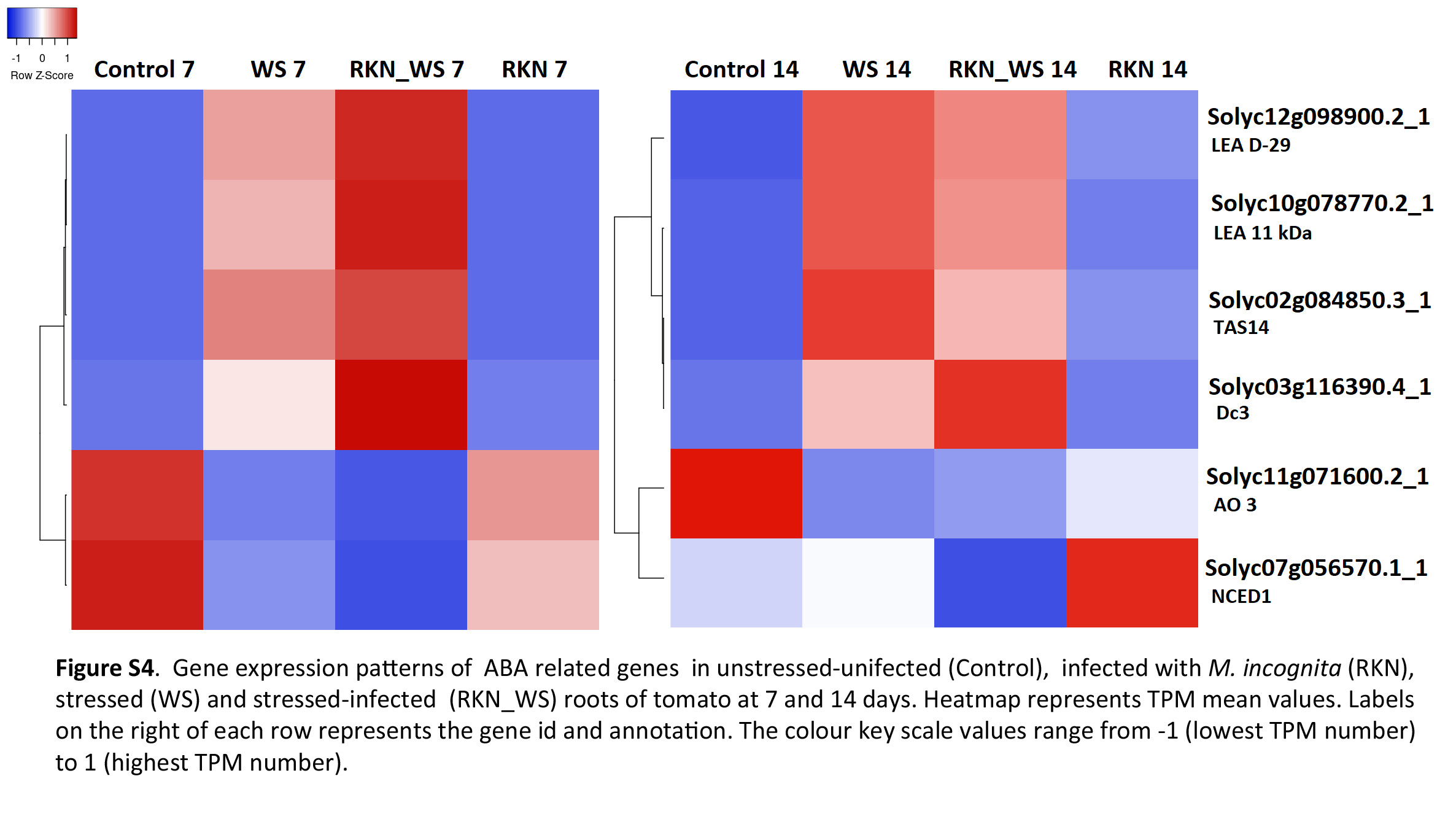

Supplement: Supplementary file 1 [file Data_Sheet_1.zip › Figure S4.JPEG]
